# Basic physical exam skills versus technology: a case of undiagnosed scleroderma

**DOI:** 10.11604/pamj.2021.40.6.31327

**Published:** 2021-09-02

**Authors:** Elias Shamieh, Husam Bader

**Affiliations:** 1 University of Jordan, Department of Internal Medicine, Amman, Jordan,; 2 Presbyterian Rust Medical Center, Internal medicine attending, Rio Rancho, New Mexico, USA

**Keywords:** Rheumatology, clinical education, abdominal pain and obstruction

## Image in medicine

A 50-year-old female with no previous known medical history was transferred to our tertiary hospital for undiagnosed chronic abdominal pain, bloating, nausea, recurrent vomiting, and extreme unintentional weight loss ongoing for the past three years. During that time, she had many emergency department visits and evaluated by multiple specialists. Previous workups include numerous CT scans, MRIs, EGDs and colonoscopies without a clear clinical diagnosis. She had been prescribed multiple courses of PPIs and antibiotics. Also, she had undergone two abdominal surgeries for suspected bowel obstruction and pseudo-obstruction. Upon encounter, the patient was noted to be severely cachectic after being on TPN for the last three months due to severe esophageal reflux, heartburn, and food intolerance. A more careful history revealed symptoms of Raynaud´s for the last 10 years. Also, when specifically asked and examined, the patient stated that her skin is hard and shiny, as if it was waxed. Based on the clinical presentation, diagnosis of CREST syndrome was suspected. That was further supported by a positive anti-centromere antibody test. Treatment options were limited due to the extent of disease progression by the time of diagnosis. Systemic sclerosis diagnosis can be challenging and requires a high level of suspicion. This case accents the gravity and value of a thorough history and physical examination as the prime foundation to reaching a diagnosis and it reminds us that sophisticated investigations are not a substitution for the essential skills of history taking and physical exam, especially in the setting of a long-standing undiagnosed illness.

**Figure 1:**
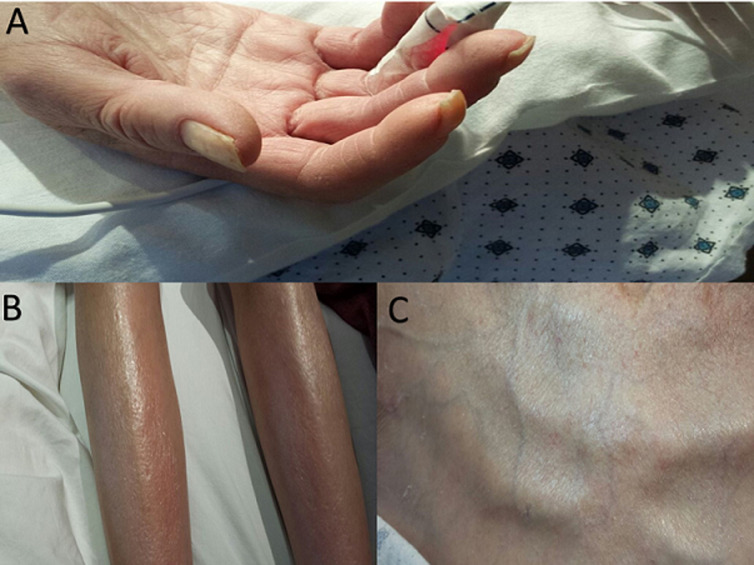
A) sclerodactyly and calcinosis of the fingers; important features of scleroderma; B) calcinosis of skin in the lower legs; C) telangiectasia

